# “Therapies Through Gut:” Targeted Drug Delivery for Non‐Gastrointestinal Diseases by Oral Administration

**DOI:** 10.1002/adhm.202403162

**Published:** 2025-03-03

**Authors:** Subarna Ray, Shehzahdi S. Moonshi, Hang Thu Ta

**Affiliations:** ^1^ School of Environment and Science Griffith University Nathan Queensland 4111 Australia; ^2^ Queensland Micro‐ and Nanotechnology Griffith University Nathan Queensland 4111 Australia

**Keywords:** gastrointestinal tract, nanoparticles, non‐gastrointestinal disease, oral administration, targeted drug delivery

## Abstract

Oral drug delivery is a promising approach for the treatment of various gastrointestinal (GI) diseases but poses significant challenges to target non‐GI diseases. The intestinal barrier forms a significant anatomical challenge to reach the target diseased site, along with various physiological challenges such as stability in the GI tract. These challenges lead to development of various targeted nanoparticles and strategies to cross intestinal barrier and protect them from harsh conditions in the GI tract, improving absorption into the circulatory system, improving bioavailability, and ensuring a regulated release. Targeting ligands such as chitosan, Butyrate (BU), and yeast capsule (YC) shows effective permeability across the intestinal epithelium. After crossing the intestinal epithelium, these targeted strategies can effectively treat various non‐GI diseases such as atherosclerosis, cancers, neurodegenerative diseases, fibrosis, and post‐traumatic osteoarthritis. However, various challenges including stability and low bioavailability still persist, which can reduce the efficacy of these therapeutics and should be considered in designing potential therapies for non‐GI diseases in the near future.

## Graphical abstract

Schematic illustration of the mechanism of targeted delivery of nanoparticles including 1) paracellular, 2) endolysosomal escape, 3) receptor mediated endocytosis, and 4) M cell mediated transport for non‐GI diseases by oral administration such as atherosclerosis, cancer, diabetes, and brain diseases.

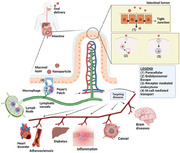

## 1 Introduction

Oral administration is one of the most prevalent and common methods of drug delivery to the diseased site for systemic treatment and for addressing GI conditions. Oral administration is the most favorable because of the various merits it poses such as convenience, non‐invasive method, and cost effectiveness.^[^ ^1^ ^]^ Patient compliance to oral formulations is typically greater compared to other parenteral routes such as intravenous, subcutaneous, and intramuscular injections, as well as to inhalation methods for asthma medications. However, oral drug delivery poses significant challenges in the delivery of drugs such as the harsh acidic environment in the stomach, which results in degradation of the drugs before reaching the target site. Various drugs with low solubility are not easily absorbed through the GI mucosa, which results in poor bioavailability, and a very small fraction of drug reaches the target site.^[^ ^2^ ^]^ Alongside challenges such as limited aqueous solubility and low permeability, presystemic clearance is also a significant factor contributing to poor oral bioavailability. An orally administered drug may be ineffective due to hydrolysis in the stomach, inconsistent delivery to the target sites in the small or large intestine for diseases such as Crohn's disease or ulcerative colitis, and the limited retention time of drugs for optimal absorption in diseases involving diarrhea.^[^ ^3^ ^]^

While many oral drug delivery systems have traditionally been designed to target local GI conditions such as gastric disorders,^[^ ^4^ ^]^ inflammatory bowel disease,^[^ ^5^ ^]^ and colon cancer,^[^ ^6^ ^]^ significant advancements in pharmaceutical technology and the physiological understanding of diseases have facilitated the development of oral nanoparticle formulations for targeting drugs to specific sites beyond the GI tract. Many barriers need to be overcome. For targeting especially non‐GI diseases by oral administration, physiological and anatomical barriers such as the intestinal mucosa act as a barrier for drugs or nanoparticles to cross the intestine and enter the systemic circulation. Anatomical barriers such as intestinal barriers need to be bypassed and enter the blood circulation. The intestinal epithelium is lined with various cell types such as epithelium, microfold (M) cells, which regulate the movement of various macromolecules, drugs across the intestine and internalized by the underlying dendritic cells to transport into the systemic circulation. The overexpression of permeability glycoproteins (P‐gps) in the epithelium of an inflamed or diseased colon would pump the drugs back into the intestinal lumen; thus, decreasing drug absorption.^[^ ^7^ ^]^ The presence of mucus on the intestinal lining hinders intracellular transport of drugs and macromolecules from the intestinal epithelial cells. The majority of mucus is composed of mucin glycoproteins, which form a viscous gel to entrap foreign particles which hinder the transport of drugs across the epithelium.^[^ ^8^ ^]^ The first pass effect of various drugs which are extensively metabolized by the liver after absorption, leading to reduced systemic availability, is an issue which reduces the bioavailability of the drug.^[^ ^9^ ^]^Physiological barriers such as large molecular size of the drugs which fail to cross the GI tract, poor solubility in the blood, and low permeability remain a drawback for oral administration to target non‐GI diseases.

Significant efforts have been made to address these challenges, driven by an enhanced understanding of the physiological characteristics of the GI tract in both health and disease. Nanotechnology has a vast application in oral drug delivery systems as nanoparticles are a promising vehicle for drug delivery enhancing oral bioavailability. Organic and inorganic nanoparticles have been extensively researched for enhancing drug tolerability, specificity, biodegradability, and targeting capability.^[^ ^10^ ^]^ Liposomes, emulsions, and nanoparticles have been utilized to enhance drug delivery of oral drugs.^[^ ^3^ ^]^ Traditional oral drug delivery methods, including conventional tablets and capsules, have limitations such as poor site‐specific drug accumulation, unfavorable body distribution, and undesirable side effects.^[^ ^11^ ^]^ Therefore, there is an immediate need for the generation of novel targeted medication system for oral administration. The use of novel drug delivery systems and nanomedicines is considered the most advanced pharmaceutical approach for enhancing oral drug delivery.^[^ ^12^ ^]^ To enhance tolerance, pharmacological specificity, biodegradability, and targeting of oral medications, both organic and inorganic nanoparticles have been investigated. For oral medications, a variety of nanocarriers such as nanoparticles, liposomes, and emulsions has been used.^[^ ^6^, ^13^, ^14^ ^]^ The majority of nanocarriers showed benefits in preserving pharmaceuticals from severe conditions in the GI tract, improving absorption into the circulatory system from the GI tract, targeting specific locations, improving bioavailability, and ensuring regulated release. Currently, oral drug delivery is mainly for systemic delivery of drug and also focused to target GI diseases.^[^ ^15^ ^]^ Targeted delivery of drugs to non‐GI diseases via oral administration is currently limited. Significant progress has been achieved in the development of oral targeted nanoparticle formulations that can transport medications precisely to locations outside of the GI system due to advancements in materials science, disease physiology research, and pharmaceutical technology.^[^ ^16^ ^]^

Based on the limitations and challenges, rapid progress in nanotechnology is made to improve bioavailability to the distal lesions of non‐GI diseases. Macrophages are attractive targets for drug delivery as they act as carrier for delivering nanoparticles to distant parts via blood circulation. Recent developments have used β1,3‐D‐glucan, a yeast composition or YC which interacts with dectin 1 present on M cells in the intestine epithelium, to prepare oral delivery systems for non GI diseases.^[^ ^17^, ^18^ ^]^ Other targeting approaches such as glycocolic acid, BU used as targeting ligands on polymeric nanoparticles for delivering drugs across the epithelium, have shown to be effective by receptor mediated endocytosis.^[^ ^19^, ^20^ ^]^ Paracellular routes by using chitosan, CSK (CSKSSDYQC) peptide have also shown to have enhanced permeability across the epithelium by opening the tight junctions (TJ).^[^ ^21^, ^22^ ^]^ Delivery to specific organs, such as the brain, lungs, or immune system, requiring overcoming barriers such as the blood–brain barrier (BBB), requires nanoparticles with surface modifications which can cross the BBB such as PEGylation, or ligands such as gambogic acid (GA).^[^ ^23^ ^]^ These modifications or advancements could be beneficial in developing efficient and effective oral treatments for non‐GI diseases.

In this review, studies on oral drug delivery administration for targeting various non GI diseases have been discussed. Various reviews elucidate the nanoparticle systems for targeting the GI tract such as colorectal cancer, inflammatory bowel disease.^[^ ^24^, ^25^, ^26^, ^27^, ^28^ ^]^ For the first time, this review comprehensively explores different targeted drug delivery systems for non‐GI diseases via oral delivery and discusses the therapeutic efficacy and various strategies involved during intestinal absorption and crossing the intestinal gut barrier. Such a comprehensive review has not been reported till date.

## 2 Strategies to Cross the Intestinal Barrier

Oral drug delivery systems are developed to treat various GI diseases and non GI diseases such as cancer, arthritis, and cardiovascular diseases by leveraging the physiological characteristics of the GI tract sites such as pH range, absorption surface area, enzymatic activity, and microbial activity.^[^ ^15^, ^29^ ^]^ Various peptides and proteins which are used as drugs exhibit poor permeability across GI mucosal and epithelial cells due to their susceptibility in a hostile GI environment, leading to reduced absorption and reduction in bioavailability by oral administration. The primary strategy for GI targeting involves prolonging intestinal residence time and enhancing absorption rates. The novel strategies to improve bioavailability include coated nanocomplex, nanocomposite carriers, calcium phosphate nanoparticles coated with polysaccharides, yeast capsules, and so on.^[^ ^30^, ^31^, ^32^ ^]^ For the purpose of targeting non‐GI diseases, various nano‐formulations are used as drug targeting; passive targeting is regulated by size for better penetration or cellular uptake to enter systemic circulation and reach the diseased site. Various challenges have been faced to deliver drug to non‐GI lesions due to the GI environment. With the advancement of technology, oral drug delivery methods are no longer limited to targeting the GI tract, allowing for gradual delivery of drugs to distant lesions associated with non‐GI disorders. Recent advances in formulation technology and a thorough understanding of disease pathophysiology have allowed researchers to successfully target administration to a range of diseases, including systemic inflammation, tumors, brain diseases, cardiovascular diseases, obesity‐related diseases, and arthritis through oral administration.^[^ ^33^, ^34^, ^35^, ^36^, ^37^ ^]^

This review discusses various strategies that are associated with nano/micro formulations to enhance the absorption and transport of drugs across the intestinal gut barrier into the systemic circulation. These approaches leverage various mechanisms to overcome the challenges posed by the intestinal epithelium and improve the overall effectiveness of drug delivery.

**Figure 1:**
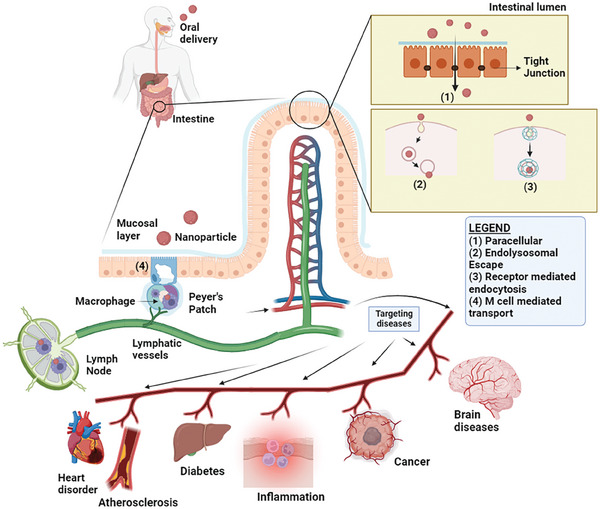
Schematic illustration of the mechanism of targeted delivery of nanoparticles including 1) paracellular, 2) endolysosomal escape, 3) receptor mediated endocytosis, and 4) M cell mediated transport for non‐GI diseases by oral administration such as atherosclerosis, cancer, diabetes, brain diseases.

Strategies in developing targeted drug delivery systems are designed to i) improve the targeting, extending in vivo residence time; thus, enhancing the therapeutic effect; ii) reduce side effects by minimizing toxicity to normal non‐targeted tissues; and iii) improve the therapeutic concentration and bioavailability; thus, decreasing the drug dosage.^[^ ^38^, ^39^ ^]^ These strategies are designed based on the physiological and biochemical pathways to enter the blood circulation as intact particles. Some drug delivery systems successfully traverse oral barriers and enter the bloodstream; while, others are digested or eliminated before reaching the portal vein. A drug delivery system which is designed to successfully and effectively cross the gut barrier would need to survive the harsh gut acidic environment, penetrate through the mucus layer present on the intestinal epithelium and internalize into the enterocytes, and avoid the hepatic clearance. In addition, a drug delivery system will likely face additional challenges such as lysosome lysis during transcellular transport and the separation of payload and the targeting ligand from the vehicles as a result of degradation or disintegration of the vehicles. Oral targeted drug delivery vehicles must preserve their structural integrity before and during absorption in order for the targeting ligand and payload to reach the circulatory system with the delivery systems.^[^ ^40^ ^]^ The strategies to achieve drug targeting via oral administration beyond the GI tract include: i) via the paracellular route by opening tight junctions (TJs); ii) developing nanocarriers capable of escaping from endo/lysosomes; iii) via transcellular pathway; iv) via receptor mediated transport; v) via transcytosis; and vi) via lymphatic transport by avoiding enterocytes and the portal vein (M‐ cell mediated transport). **Figure** **1** summarizes the fate of nanoparticle delivery via the different strategies discussed above for oral administration for targeting non‐GI diseases. Various reported studies using different approaches for drug delivery systems utilizing nano/microparticles to get through the intestinal barrier are tabulated in **Table** **1**.

**Table 1:** Various strategies for nanoparticles to target and cross the GI tract.

Strategy	Responsible material	Mechanism	Effectiveness	Reference
Paracellular transportation (opening of TJs)	Chitosan	Chitosan is associated with translocation of JAM‐1 (a TJ protein) in the cell–cell junction.	In vitro assay has shown opening of TJ with chitosan treatment after 30 min.	[21]
	CSK peptide	CSK targets goblet cell present in the intestinal epithelial. As CSK is conjugated with trimethyl chitosan (TMC), interaction between positive charge chitosan (TMC) and negative charge in the endothelial cavity facilitates the paracellular transport.	CSK‐TMC has shown higher permeability across the epithelium than TMC.	[22]
	Polyamidoamine (PAMAM)	PAMAM modulate negatively charged TJ proteins due to the presence of positive charged amino group.	PAMAM has enhanced paracellular transport and improved permeability.	[42]
Endo lysosomal escape	EGP peptide with N terminal cysteine group (KRKKKGKGLGKKRDPCLRKYKC)	EGP peptide has high affinity to heparan sulfate proteoglycans and escapes the endo lysosomal pathway	Increase in transcytosis efficacy by 4.2 fold	[46]
	Hemagglutinin‐2 (GLFEAIEGFIENGWEGMIDGWYG, HA2) peptide	Hemagglutinin‐2 destabilizes the membrane of endosomes and escapes endosome entrapment.	Hemagglutinin 2 effectively transports the insulin loaded nanoparticles across the intestinal epithelium for 2.19 fold than non‐targeted counterparts.	[45]
	Metformin and Hemagglutinin‐2	Escapes the entrapment by late endosome, which converts into lysosome	Improved transcytosis by 13% when compared to metformin and hemagglutinin‐2 alone	[44]
Receptor‐mediated transport	Butyrate (BU)	BU binds to MCT1 receptor on the intestinal epithelium	Improved endocytosis and uptake by 0.8 fold.	[19]
	Arginine–glycine–aspartic acid (RGD peptide)	RGD targets β1 integrins on intestinal epithelium	Increased cellular permeability and higher uptake efficiency in all liposomal formulations in co‐culture systems of Raji‐B and Caco‐2 cells	[49]
	Folic acid (FA)	FA binds to folate receptors present in the intestine epithelium	Higher penetration/uptake by 0.52‐fold than non‐targeted	[50]
	IgG Fc domain‐binding peptide (FcBP peptide)	FcBP peptide binds to Fc receptor (FcRn) overexpressed in the intestinal epithelium	Improved endocytosis by 1.67‐fold in the epithelium	[52]
	Glycocolic acid (bile acid)	Glycocolic acid binds to apical sodium‐dependent bile acid transporter (ASBT) present on the enterocyte of the ileum	Improved penetrance/uptake by threefold via the ileum into the lymphatic and blood circulation	[20]
	Deoxycholic acid (DA)	DA binds to apical sodium‐dependent bile acid transporter (ASBT)	Improved penetrance/uptake by onefold	[54]
	Angiopep‐2	Angiopep 2 binds to low‐density LRP‐1 present on the intestine	Improved penetrance/uptake by onefold	[55]
	Gambogic acid (GA)	GA binds to the transferrin receptors present on the intestinal epithelium	Enhanced transport across the intestinal epithelium by three fold	[51]
M cell mediated transport	Yeast capsule (YC)	β glycan present on yeast membrane targets dectin‐1 present on macrophage	Higher accumulation and penetration by 2.5‐fold in PP, MLN, thymus region	[67]
			Higher penetration in PP by 0.38‐fold than free group (without yeast)	[18]
			Higher accumulation in PP	[17]
			Threefold increase in penetration in PP	[88]
			Higher accumulation of nanoparticles in PP	[78]
	Mannose	Endocytic pathway mediated by mannose receptors on the M cell	Enhanced accumulation at the PPs, mainly through M cells and targeting of antigen presenting cells	[81]
	Tomato lectins	Lectins recognize the glycolipids or polysaccharide‐protein complex present on M cells to mediate endocytosis	Enhanced accumulation of nanoparticles in PP and MLN compared with unmodified nanoparticle	[77]
	Aptamer	Aptamer targets the M cells present on the intestinal epithelium	Improved intestinal absorption of aptamer‐tagged liposomes in the PP in vitro	[80]
	*U. europaeus* agglutinin 1	*U. europaeus* agglutinin 1 targets fructose receptors present on the M cells	Improved delivery of insulin across PP and intestine by 4.1‐ and 2.6‐fold, respectively	[79]

### 2.1 Paracellular

**Figure 2:**
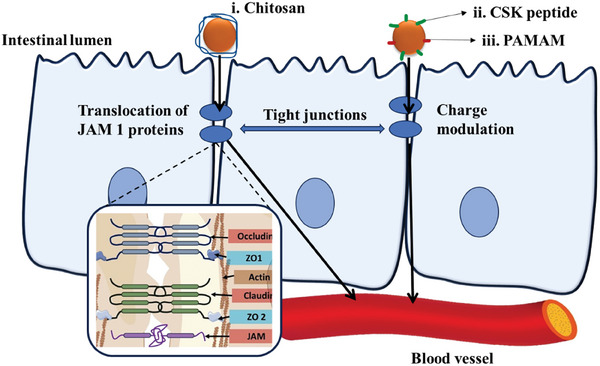
Schematic illustration of the mechanism of action for transport of nanoparticles with ligands: 2‐i) chitosan, 2‐ii) CSK peptide, and 2‐iii) PAMAM by paracellular methods, which involve the interaction of the ligands with the tight junction proteins, where opening the junction proteins allows the transport across the epithelium.

The paracellular pathway is one possible method for oral targeted medication delivery. Paracellular transport mechanism is a passive approach across the gradient developed by primary and secondary transport proteins in transcellular pathway. Through this pathway, drug carriers bypass the cell, thereby avoiding intracellular degradation. Certain compounds, especially polycations, have demonstrated the ability to temporarily open the TJs between adjacent enterocytes, facilitating the penetration of small nanoparticles into capillaries.^[^ ^41^ ^]^ Polyamidoamine (PAMAM) polycation exhibits a great potential to open TJs by enlarging the pore radius and improving the permeability of hydrophobic and hydrophilic small molecules as well as hydrophilic macromolecules through the small intestines. TJs are a complex of negatively charged proteins including occludin, claudins, junctional‐associated membrane protein (JAM), and zonula occludens proteins (ZO‐1, ZO‐2). PAMAM dendrimers are positively charged due to the presence of amine groups and interact with negatively charged TJ proteins, leading to conformational changes and transient opening of the junctions.^[^ ^42^ ^]^ In various intestinal segments, the transport efficiency of doxorubicin when complexed with PAMAM is enhanced by four to seven times in comparison with the free drug, leading to a 200‐fold increase in bioavailability.^[^ ^43^ ^]^ Sonaje et al. demonstrated that chitosan enhanced TJ opening and paracellular transport. The positively charged chitosan interacted with negatively charged TJ protein and regulated the permeability of various ions and molecules across the epithelium. This underlying mechanism has been linked to transmembrane TJ protein (JAM‐1) translocation, which results in reversible TJ disruption, making chitosan an effective permeation enhancer.^[^ ^21^ ^]^ In another study, peptide CSKSSDYQC (CSK) coupled with N‐trimethyl chitosan was employed, whereby the former targeted intestinal epithelial cells to penetrate the intestinal mucosa and the latter reversibly opened TJs, aiming to improve drug uptake and efficacy.^[^ ^22^ ^]^ **Figure** **2** describes the methods of transport of the nanoparticles with ligands as mentioned above by paracellular methods.

### 2.2 Endolysosomal Escape

**Figure 3:**
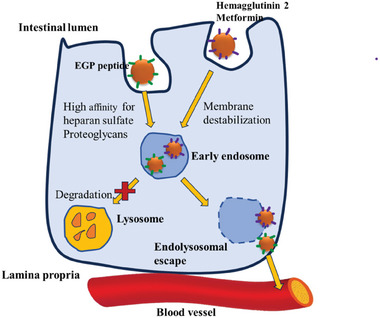
Schematic illustration of the mechanism of action for transport of nanoparticles with ligands (EGP peptide, hemagluttinin 2, and metformin) by endolysosomal escape involving attachment of ligand to receptors, membrane destabilization, forming early endosome, and escaping the formation of lysosome.

Endolysosomal escape is a mechanism to divert from the endosomal vesicles which contain acid hydrolases which can inactivate various therapeutics. This escape is crucial for the translocation of drug vehicles into systemic circulation, especially when endocytosis and transcytosis cannot be avoided as it prevents lysosomal breakdown of payloads and materials. Combining nanoparticles with an endo/lysosomal escape agent can improve the oral absorption and systemic exposure of payloads. Nanocarriers paired with agents such as hemagglutinin‐2 and metformin have been shown to inhibit endolysosomal entrapment and increase basolateral exocytosis.^[^ ^44^, ^45^ ^]^ Zheng et al. developed an innovative nanoparticle strategy (size 100–115 nm, PDI 0.22, charge +15.5 mV) capable of successfully delivering proteins and biomacromolecules orally by controlling the transcytosis pathway.^[^ ^46^ ^]^ The EGP peptide (KRKKKGKGLGKKRDPCLRKYK) ligand‐modified nanoparticles were able to avoid lysosomal trapping and internalized by caveolae‐mediated endocytosis, facilitating apical‐to‐basolateral transcytosis. EGP peptide bypasses the endolysosomal pathway without disrupting the endosomes. This is mediated due to the presence of highly cationic charge of EGP, which acts as a “proton sponge” inside endosome attracting protons as the endosome acidifies. This effect, often facilitated by polycationic molecules, leads to an influx of counterions, causing osmotic swelling of the endosome. This results in increased osmotic pressure, leading to the rupture of endosomal membrane, allowing the EGP peptide to escape into the cytosol before the endosome matures into a lysosome.^[^ ^47^, ^48^ ^]^ Therefore, endolysosomal escape method has shown to have some relevance for oral targeted drug delivery. **Figure** **3** describes the methods of transport of the nanoparticles with ligands as mentioned above by endolysosomal escape.

### 2.3 Receptor Mediated Transport

Receptor mediated endocytosis involves the uptake of a large variety of different cargoes which interact with receptors from the plasma membrane into the cell. Generally, clathrin and caveolae mediated endocytosis is the most widely studied. Nanoparticles functionalized with specific ligands targeting receptors on intestinal cells facilitate their transport across the epithelium via endocytosis. During this process, the nanoparticles are enclosed within vesicles, enabling the delivery of drug‐loaded nanoparticles into the systemic circulation, thereby improving overall drug delivery efficiency.

Poly(lactide‐co‐glycolide) (PLGA)/lipid hybrid nanoparticle functionalized with BU has shown better cellular uptake and endocytosis by 0.8‐fold in Caco2 and mouse hepatoma (Hepa1‐6) cells than the non‐targeted nanoparticles (NP). The molecular interaction between BU and monocarboxylate transporter 1 (MCP1) on Caco2 and Hepal‐6 of the intestinal epithelium facilitated endocytosis, improved permeability, and enhanced oral absorption.^[^ ^19^ ^]^ Another study investigated the use of follicular stimulating hormone (FSH) loaded nanostructured lipid carriers labeled with arginine–glycine–aspartic acid (RGD targeting peptide) for female infertility, having shown increased cellular permeability and higher uptake efficiency in all liposomal formulations in co‐culture systems of Raji‐B and Caco‐2 cells. The interaction between RGD targeting peptide and β1 integrins present on intestinal epithelium enhanced the endocytosis process of nanoparticle transport across intestinal epithelium.^[^ ^49^ ^]^

Guo et al. studied the in vitro cellular uptake and transport of lipid‐PLGA‐based nanoparticles modified with folic acid (FA), which binds to folate receptors present on the GI tract for naringenin delivery, enhances the transport of nanoparticles across the intestinal epithelium via the gut associated lymphoid tissue (GALT) pathway, and targets atherosclerotic plaque. The nanoparticles showed higher penetration by 0.52‐fold than non‐targeted nanoparticles, and cumulative naringenin transport increased over time. The FA‐modified NPs showed greater cellular uptake in Caco‐2 and HT‐29 cells than non‐targeted NPs due to better compatibility and active targeting.^[^ ^50^ ^]^ The study of GA conjugated PLGA nanoparticles uptake in Sprague Dawley rats revealed that its uptake was concentration‐dependent, suggesting active targeting of GA to transferrin receptors on intestinal epithelium, which enhanced the delivery of PLGA nanoparticles across epithelium and entered systemic circulation to reach the liver. Fluorescence analysis showed targeted NPs crossed villi and entered lamina propria.^[^ ^51^ ^]^

**Figure 4:**
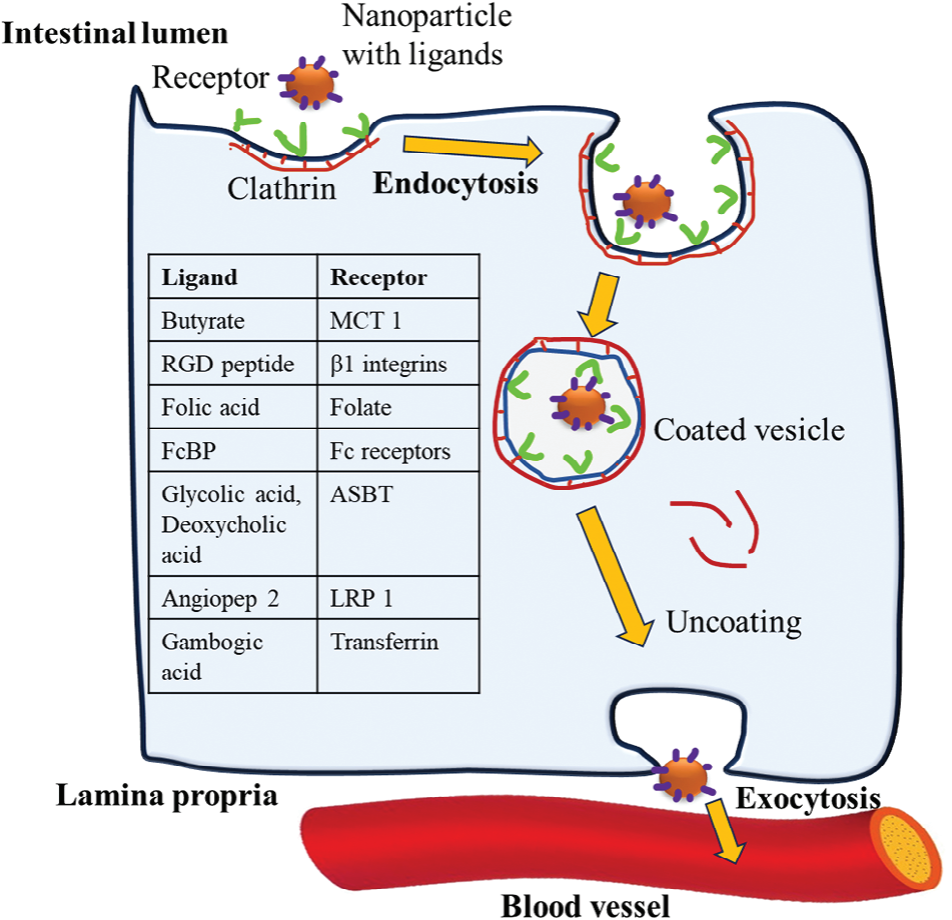
Schematic illustration of the mechanism of action for transport of nanoparticles with ligands by receptor mediated endocytosis which involves the ligand attachment, endocytosis, transport across the cell, and exocytosis.

PLGA‐encapsulated lipid shells modified with IgG Fc domain‐binding peptide (FcBP peptide) to target intestinal epithelium for liver fibrosis treatment were studied. Different rigidities were used, including soft (consisting of only fattened lipid shell), semi, and stiff NPs (PLGA core packed in lipid shell). The FcBP modification improved mucosal permeability, fluorescence, and mucus permeation. Semi NP showed the highest permeation; while, FcBP modification significantly improved endocytosis by 1.67‐fold in the epithelium. The molecular interaction between FcBP peptide and Fc receptors present on intestinal epithelium enhanced the trans epithelium transport of nanoparticles across the intestinal epithelium and delivery of nanoparticles into the systemic circulation.^[^ ^52^ ^]^ The efficacy of the receptor mediated transcytosis was dependent majorly on the binding affinity and specificity of a ligand to its particular receptor, which subsequently mediated the endocytosis process. From the above results, it is evident that FcBP peptide modification has shown a higher penetration in the intestinal epithelium, which binds to the Fc receptors present in the epithelium. Bile acids or bile salts act as mediators for receptor mediated endocytosis by targeting apical sodium‐dependent bile acid transporter (ASBT) present on the enterocyte of the ileum and absorbed into the systemic circulation.^[^ ^53^ ^]^ The use of glycocolic acid and deoxycholic acid has shown an enhanced permeation via the ileum and transported to the systemic circulation.^[^ ^20^, ^54^ ^]^ Other peptides as ligands such as angiopep‐2 have also shown enhanced penetrance into the enterocytes present on the intestinal epithelium by binding to their respective receptors such as low‐density lipoprotein receptor‐related protein 1 (LRP‐1) and epidermal growth factor receptors.^[^ ^55^ ^]^ **Figure** **4** describes the methods of transport of the nanoparticles with ligands as mentioned above by receptor mediated transport.

Generally, it was observed that receptor mediated transport to cross intestinal barrier has an efficient penetration potential to enter the systemic circulation, as evident by high fluorescence signals after transport, and is also dependent on the number of receptors present on the intestinal epithelium for better targeted transport across the intestines.

### 2.4 M Cell Mediated Transport

**Figure 5:**
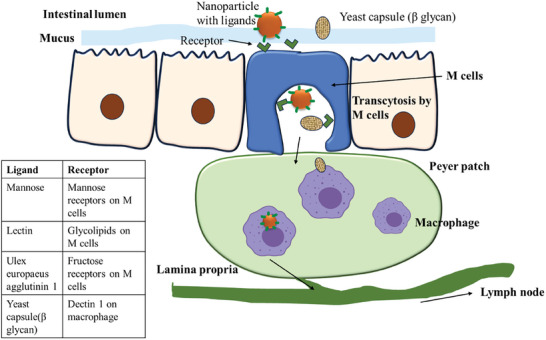
Schematic illustration of the mechanism of action for transport of nanoparticles with ligands/yeast capsule by M cell mediated transport, which involves the ligand attachment on M cell, transcytosis by M cell, macrophage uptake in peyer patch, and transport to lymph node.

M cells or microfold cells which form a gateway for various nanoparticle transports to the Peyer's Patch (PP) found in the small intestine is one of the promising strategies for nanoparticle transport. M cells also form a part of the GALT which serves as a pathway for various antigens to reach the intestinal immune system via the follicle associated epithelium.^[^ ^56^ ^]^ The M‐cell‐mediated pathway is utilized for delivering nanoparticle drugs because M cells have advantages over enterocytes, including reduced intracellular enzymatic activity, a much thinner mucus layer, and a less dense glycocalyx.^[^ ^57^ ^]^ These elements facilitate access and intracellular trafficking. After being taken up by M cells, nanoparticles may either be trapped by immune cells present in the PP, which facilitates oral vaccines, or they can be passively targeted and then delivered systemically.^[^ ^58^, ^59^ ^]^ Briefly, the specialized M cells present on the intestinal epithelium act as gatekeepers to enter the systemic circulation. The nanoparticles first adhere to the apical surface of cells via specific ligand–receptor interactions or non‐specific adhesion. The M cells have sparse microvilli and a thin glycocalyx, allowing easier access for nanoparticles compared to regular enterocytes. After adhesion, the nanoparticles are internalized into vesicles by various means such as Clathrin‐mediated endocytosis, Caveolin‐mediated endocytosis, or macropinocytosis and also depend on nanoparticle size, surface properties, and targeting ligands. After endocytosis, nanoparticles are transported across the M cell in vesicles and exocytosed at the basolateral membrane of the M cell into the subepithelial dome region into the PP. Immune cells (e.g., dendritic cells, macrophages) present in the PP can process the delivered nanoparticles for initiating a systemic delivery.^[^ ^60^ ^]^ Interestingly, whilst NPs that are captured by macrophages could render the targeting of NPs to be inactive at the diseased site, all studies have shown good therapeutic outcome with this approach. **Figure** **5** describes the methods of transport of the nanoparticles with ligands as mentioned above by M cell mediated transport. It is estimated that this pathway accounts for ≈0.2–70% of nanoparticle delivery efficiency range‐wise depending on the factors such as particle size and surface modifications. Various physicochemical parameters, size, surface modifications, and polymer properties have an effect on the efficiency of M cell mediated transport

#### 2.4.1 Size

The size of the nanoparticle is a significant factor in controlling the transport across epithelial layer by M cells. Generally, the size range below 500 nm, especially smaller than 200 nm, can easily penetrate across mucus and be taken by enterocytes, finally transporting in portal veins. The preference of uptake by M cell into Peyer's Patch and the fate of transport by lymphatics are not only dependent on the size but also on the material used for nanoparticle. In vivo studies have shown that polycaprolactone (PCL) nanoparticles of larger size (600–2000 nm) could be transported more efficiently by M cells than by smaller nanoparticles, that is, 50 and 200 nm.^[^ ^61^ ^]^ Various other polymers such as polystyrene, poly(methyl methacrylate), poly(hydroxybutyrate), poly(dl‐lactide), poly(l‐lactide), and poly(dl‐lactide‐co‐glycolide) (<1 µm) have shown efficient translocation across M cells but those more than 5 µm remain entrapped in the PP and not transported to the lymph.^[^ ^62^ ^]^ Other studies using ultrafine amorphous particles (UAPs) of cyclosporin A (CsA) have shown an increased preference in transcytosis for 550 and 1100 nm as compared to 250 nm, with elevated levels in jejunum and ileum proving to be effective in lymphatic transport.^[^ ^63^ ^]^ Quercetin hybrid nanocrystals have also shown enhanced transcytosis and cellular uptake of nanoparticle of size 550 and 1100 nm than its smaller counterpart, that is, 250 nm.^[^ ^64^ ^]^ Similarly, ultrafine amorphous particles (UAPs) of cyclosporin A (CsA) have shown an increased preference in transcytosis for 550 and 1100 nm, as compared to 250 nm, with elevated levels in jejunum and ileum proving to be effective in lymphatic transport.^[^ ^63^ ^]^ Titanium dioxide nanoparticles (18 nm) were also tested for transport by M cells, but nanoparticles were not observed in the intestinal or PP patch.^[^ ^65^ ^]^ Thiol organosilica nanoparticles have shown a size dependent absorption with the higher absorption by the smallest size, that is, 95 nm and lowest for 1050 nm.^[^ ^66^ ^]^ It is noteworthy that yeast or yeast derived microcapsules which are also in the size range of 3–5 µm have displayed effective transport via M cells and promising therapeutic outcome in atherosclerosis and cancer in in vivo models.^[^ ^17^, ^67^ ^]^ Ex vivo study has shown that smaller polystyrene nanoparticles (50, 200 nm) were taken up by PP but were not transported in an ex vivo porcine intestinal model.^[^ ^68^ ^]^ However, titanium dioxide (12, 130 nm) has shown smaller size (12 nm) transported across ex vivo tissue, but 130 nm was found less in the ileum epithelium.^[^ ^69^ ^]^

In vitro studies were also performed to study the transport of various nanoparticles across the M cell model. Typically, Caco‐2 cells cocultured with cells from the lymphoreticular system (mostly Raji B cells) interact via soluble factors and acquire an M cell like transcytosis activity.^[^ ^70^ ^]^ As Caco 2 cells do not have the ability for mucus production, often HT29 cells, derived from a human colorectal adenocarcinoma, functionally mimic goblet cells and actively secrete mucus in vitro. This model however, does not fully recapitulate the barriers hampering the in vivo uptake across the intestinal epithelium; and thus, overestimate the uptake and transport.^[^ ^71^ ^]^ The use of polystyrene nanoparticle for uptake and via M cell model have all shown consistent results with the inverse correlation of transport/permeability with size. The smaller size has shown highest transport as compared to its larger counterparts.^[^ ^68^, ^72^, ^73^, ^74^ ^]^ Therefore, it can be observed that the permeability of nanoparticles across M cell in vitro is inversely proportional to the nanoparticles’ size irrespective of any size limit. Banerjee et al. performed a comparative analysis of various sizes for permeability across M cell in vitro, and an inverse correlation of transport and size was observed with uptake of 50 > 200 > 500 > 1000 nm.^[^ ^73^ ^]^ Other nanoparticles such as TiO_2_‐NP (18 nm) and nanoemulsions (80, 550, 1000 nm) had shown a similar correlation with the highest permeability for smaller sized nanoparticles in vitro.^[^ ^69^, ^75^ ^]^ **Table** **2** tabulates the in vivo, ex vivo, and in vitro studies on the dependence of size with permeability across intestinal epithelium by M cell mediated transport. The preference for small sized nanoparticles to be transcytosed by M cells and enterocytes is based on preference over larger nanoparticles and demonstrates higher intestinal transport. However, the exact reason for this observation or discrepancy on why certain sizes show effective transport by M cells while others don't has not been addressed clearly. In vitro and in vivo studies have indicated varying results with respect to size of the micro/nanoparticle. However, comparing findings across various studies proves challenging due to differences in animal models used, as well as variations in materials employed and measurement techniques.

**Table 2:** Studies in the last 10 years reporting the absorption of particles by Peyer's patches and microfold cells.

Model	Particle with size	Conclusion	Reference	Publication year
In vivo and ex vivo models of Peyer's patches and microfold cells				
Sprague–Dawley (SD) rats	50, 200, 600, and 2000 nm Polycaprolactone	Large nanoparticles of 600–2000 nm were transported more via the lymphatics than the smaller ones (50 and 200 nm)	[61]	2018
BALB/c mice	1–10µm Polystyrene, poly(methyl methacrylate), poly(hydroxybutyrate), poly(dl‐lactide), poly(l‐lactide), and of poly(dl‐lactide‐co‐glycolide)	Nanoparticles below 1 µm are taken up by M cells and transported toward the basal medium; while, particles larger than 5 µm are taken up by M cells but remain entrapped in Peyer's patches	[62]	2018
Sprague–Dawley (SD) rats	250, 550, and 1100 nm Ultrafine amorphous particles (UAPs) of cyclosporin A (CsA)	There is increased translocation of 550 nm UAPs via the ileum promoted by the M cell‐mediated transcytosis. 250 nm UAPs are internalized more than the larger particles.	[63]	2018
Sprague–Dawley (SD) rats	280, 550, and 1100 nm Quercetin hybrid nanocrystals	Higher transport for 550 and 1100 nm in ileum than in jejunum; cellular uptake higher for 550 nm than 550 and 1100 nm	[64]	2018
Sprague–Dawley male rats (18 nm)	18 nm Titanium dioxide nanoparticles (TiO_2_ NPs)	No NPs were observed in intestinal or Peyer's Patches’ cell	[65]	2014
C57 BL/6J mice	95, 130, 200, 340, 695, and 1050 nm Thiol organosilica	Size dependent absorption (high percentage of smaller NPs in PPs), with the highest percentage for 95 nm and the lowest for 1050 nm in the subepithelial dome on intestine	[66]	2012
Male Sprague–Dawley (SD) rats	Nanoemulsion (80, 550, and 100 nm)	80 nm nanoemulsions (NEs) were more pervasively distributed into enterocytes and retained higher in the small intestine than large NEs (550 and 1000 nm)	[75]	2017
Rat	3–5 µm YC	Efficient transport of yeast capsule across the M cell in Peyer's patches	[17, 67]	2020, 2017
Ex vivo porcine intestine in using chamber	50, 200 nm polystyrene nanoparticles	Smaller NPs (50, 200 nm) taken up by PP but not transported	[68]	2014
Ex vivo mouse ileum	12 and 130 nm Titanium dioxide	12 nm particles transported across ex vivo tissue; 130 nm particle found in lower conc. in the ileum epithelium	[69]	2014
In Vitro Models of M‐Like Cells				
Caco‐2, co‐culture HT29‐MTX, and tri‐culture Raji B cells	1, 4, and 10 µm Polystyrene microparticles	Enhanced uptake and transport of 1 µm (5.8% of the total NP) compared to 4 and 10 µm which corresponds to up to 3.8% and 0.07%, respectively	[72]	2019
Caco‐2/HT‐29/Raji‐B	Polystyrene microparticles 50, 200, 500, and 1000 nm	Size dependent absorption: inverse correlation with size, that is, highest for the smallest nanoparticle 50 > 200 > 500 > 1000 nm	[73]	2016
Caco‐2/HT29‐MTX/B cells	50 and 200 nm Polystyrene nanoparticles	Higher permeability for the smaller nanoparticles, that is, 50 nm than the larger counterparts, that is, 200 nm	[68]	2014
Caco‐2 inverted+ Raji B cells	500 nm, 1 µm Polystyrene particles	Higher permeability for 500 nm than 1 µm (3.6‐fold)	[74]	2017
Caco‐2/RajiB coculture	18 nm TiO_2_‐NP	Higher transport in the M cell model as opposed to Caco 2 cells alone	[69]	2015
Caco‐2/HT29‐MTX model	Nanoemulsion (80, 550, and 1000 nm)	Negative correlation between particle size and cellular uptake transport Smaller size, that is, 80 nm, has shown higher penetrance than 550 and 1000 nm	[75]	2017

#### 2.4.2 Surface Modifications

With application of various surface modifications in the form of ligands, surface coating has been explored for better transport through M cells. Chitosan, due to its mucoadhesive properties, adheres to the mucosal surface, increasing the residence time of particles at the M cell‐rich PP. Chitosan derivates have also shown enhanced absorption of insulin in colon by M cell mediated transport.^[^ ^76^ ^]^ Chitosan in combination with antigens can facilitate antigen presentation to immune cells, boosting vaccine or therapeutic efficacy. Positively charged compounds such as chitosan improve the adhesion with negatively charged glycocalyx and mucus layer and enhance the transport via M cells.

Pan et al. explored the application of tomato lectins as ligands on nanoemulsions to target M cells. Lectins are known to bind directly and specifically to glycolipids or polysaccharide–protein complex of intestinal epithelial cells rather than to mucus, unlike macromolecule. In addition, lectins are also responsible for triggering the active transport process of endocytosis and convey the biological signals to the cells. A prolonged interaction between lectin coated nanoemulsion and mucus is observed, leading to an increase in uptake rate across intestinal epithelial cells.^[^ ^77^ ^]^

Liu et al. developed innovative layer‐by‐layer assembled lutein nanoparticles self‐assembled with chitosan and 3‐boronobenzoic acid modified yeast β‐glucan, which targeted the dectin‐1 receptors in M cells, for the delivery of phycocyanin, an effective intervention for dry eye disease. This innovative approach could effectively and specifically target M cells in vitro, significantly improve the bioavailability, effectively relieve dry eye disease, and alleviate corneal damage.^[^ ^78^ ^]^

Targeting ligands such as *Ulex europaeus* agglutinin 1 (UEA‐1, a representative lectin), targeting the fructose receptors on M cells, have improved the insulin uptake in PP and intestine by 4.1‐ and 2.6‐fold, respectively.^[^ ^79^ ^]^ As explained above, aptamers were also effective ligands increasing the transport efficiency of exenatide by twofold in M cells and showed better absorption in PP.^[^ ^80^ ^]^ In a recent study, aptamers were used as a targeting ligand on liposomes for the delivery of exenatide across the intestinal epithelium. Aptamers are short ssDNA, RNA, or modified nucleic acids known for its high binding affinity and specificity to M cells. Liposome–aptamer complex (5′‐CGAGGGGCACCCTCGACCCGTCCCGACAGGATTTGGCGCAGGGGG‐3′) has shown a significantly high intestinal absorption in PP by twofold and M cells‐specific targeting capacity and could be a promising M cell targeted delivery system for oral delivery of macromolecules.^[^ ^80^ ^]^

A study examined the absorption and translocation of FA‐labeled PLGA‐lipid NP in the GI tract of Sprague–Dawley rats. The ileum, containing the PP, showed improved cellular uptake by 1.7 times due to enhanced FA targeting.^[^ ^50^ ^]^ This was attributed to the presence of lipid bilayer and active targeting of external ligand FA. Transepithelial transport was facilitated by M cells and macrophage phagocytosis. Mannose and RGD pepide also showed capability to enhance PP uptake. Eudragit L100‐coated mannosylated chitosan nanoparticles showed greater penetration into the ileal region of Sprague–Dawley rats, primarily localized in protrusion of the PP.^[^ ^81^ ^]^ Eudragit coating was employed to protect the nanoparticles; while, mannose was for targeting dendritic cells. Mannosylated NPs which target antigen presenting cells have shown higher efficacy in delivering antigens to the effective sites due to higher fluorescence signal localized in the PP region as compared to non‐mannosylated group. The mannose receptors are expressed widely in macrophages and dendritic cells, and the endocytic pathway mediated by mannose receptors can be 100 times more significant than other routes.^[^ ^82^ ^]^ RGD peptide conjugated liposomes have shown a higher permeability by 0.24‐fold, as compared to non‐conjugated liposomes due to the binding to β‐integrins of M cells and facilitating the transport to lymphatic system.^[^ ^49^ ^]^

**Figure 6:**
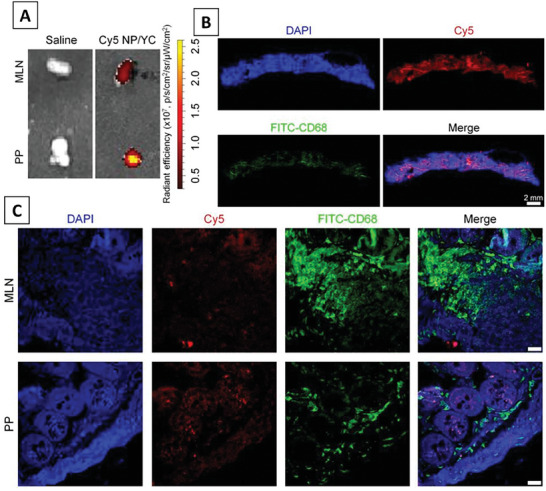
Fluorescence imaging of intestinal barrier oral delivered therapy and accumulation and penetrance in intestinal organs: in vivo penetration studies of YC across the intestinal epithelium to deliver cisplatin to tumor: A) Ex vivo images of PP and MLN with Cy5 NP/YC after oral delivery in mice with A549 xenografts indicating significant penetrance of Cy5 NP/YC in both lymphocytic tissues, that is, MLN and PP related to intestinal absorption and B) MLN isolated from Cy5 NP/YC‐treated mice showing fluorescence reveal the presence of considerable Cy5 signal; the overlapping red fluorescence of Cy5 with green fluorescence of CD68+ macrophages indicates successful uptake and transport of Cy5 NP/YC in MLN. C) Confocal microscopy images of MLN and PP sections show the presence of Cy5 signal in both MLN and PP sections, which are partially colocalized with CD68+ macrophages. Reproduced from the CC‐BY open access publication (Creative Commons Attribution 4.0 International licence).^[^ ^18^ ^]^ Copyright 2019, IVY spring.

Yeast capsules (YC) have been employed in many studies for targeting M cells due to the presence of β glucan, which targets dectin 1 on M cells, aiding the process of transcytosis across the M cells. YC tagged with Cy7.5 was developed to target plaque via oral administration, showing higher accumulation in PP, mesenteric lymph nodes (MLN), thymus region, spleen, liver, and stomach, compared to free Cy7.5 group.^[^ ^83^ ^]^ In another study of YC, using quantum dots for intestines targeting has shown that YC was internalized into CD68 macrophages in mesenchymal lymph node and PP, absorbed by PP with 2.5‐fold, as compared to free group, ferried by M cells, endocytosed by macrophages, and carried to lymphatic tissue and MLN.^[^ ^67^ ^]^ In another study, Cy5‐loaded YC was administered to A549 xenografts on the PP and MLN, revealing that Cy5 signals from MLN and PP overlapped and partially colocalized with macrophages, suggesting YC absorption via lymphatic systems.^[^ ^18^ ^]^ **Figure** **6**A–C depicts the effective penetrance and uptake of Cy5‐loaded YC across the intestinal epithelium and colocalization in MLN and PP. b‐1,3‐D‐glucan porous microcapsule (GPM) enveloping folate‐conjugated chitosan‐functional liposome (FCL) (FCL@GPM) has also shown higher penetration into PP than ileum and jejunum, indicating that the NPs permeated through mucus layer, efficiently uptaken by intestinal epithelium and accumulated inside PP.^[^ ^17^ ^]^

#### 2.4.3 Hydrophobicity

The surface hydrophobicity/hydrophilicity is an important parameter which could affect the absorption of nanoparticles across the intestines. Due to the lipid bilayer composition of the cell membrane and its inherent lipophilicity, nanoparticles with higher hydrophobicity tend to be more readily internalized by cells.^[^ ^84^ ^]^ Rieux et al. observed that nanoparticles with greater hydrophobicity enhance uptake by M cells, facilitating more efficient oral absorption through transepithelial transport.^[^^]^ Further, hydrophobic polymers, including polystyrene, polymethyl methacrylate, polyhydroxybutyrate, and glycolic acid‐based nanoparticles, show improved absorption in intestinal PP compared to the less hydrophobic lactic acid‐based polymers. Hydrophobic particles exhibit an absorption capacity ≈100 times greater than that of hydrophilic cellulose polymers.^[^ ^86^ ^]^ Higher hydrophobicity enhances transport across epithelial cell layers but impedes mucus layer penetration as surface hydrophilicity is essential for mucus permeation. To address this, Cui et al.^[^ ^84^ ^]^ developed nanoparticles with a balanced hydrophilicity/hydrophobicity by adjusting the ratio of hydrophilic *N*‐(2‐hydroxypropyl) methacrylamide (HPMA) and hydrophobic FA analogues. Nanoparticles coated with 20% HPMA–cetyl methacrylate copolymer (NPs‐C16 (20%)) demonstrated the optimal hypoglycemic effect in vivo. This highlights the importance of achieving a proper hydrophilicity/hydrophobicity balance for effective nanoparticle design.

#### 2.4.4 Summary Note on Strategies for M Cell Mediated Transport

One of the limitations of M cell mediated transport is the lower prevalence of M cells on the intestinal epithelium, constituting less than 1% of the absorptive intestinal epithelium's total surface area.^[^ ^87^ ^]^ The percentage of uptake also depends on the in vivo model because rabbit PP tissue has significantly more M cells population than rats (46% compared to 10% respectively).^[^ ^71^ ^]^ The choice of nanoparticles with respect to the size, material, shape, and charge also forms an important parameter.

From the above studies employing various particles such as YC, polymeric nanoparticles for M cell mediated transport, it can be inferred that most polymeric nanoparticles would need additional receptors for M cell targeting which could enhance easy uptake of these nanoparticle into PP for systemic circulation. Polymeric nanoparticles without any targeting ligand for M cell receptors have shown lower permeability and penetration compared to its other counterparts with the ligand. YC has been predominantly employed for M cell mediated delivery in many studies. It is a potential targeting carrier for drugs/nanoparticles, which is easily uptaken by the M cells due to the natural presence of β‐integrins which act as a binding site for dectin‐1 present on the yeast microcapsule. YC does not need any other additional receptor for targeting and facilitates the penetration into the PP for transport to the lymphatic systems. Based on the current findings, modification of polymeric or synthetic nanoparticle with YC fragment coated on the surface would enhance the penetrance and delivery across the intestinal epithelium, which could be explored more in the future.

## 3 Strategies to Target Nanoparticles to Diseased Site

### 3.1 Atherosclerosis

PP route and lymphatic system have been employed to target drug to atherosclerosis via oral administration. Atherosclerosis is caused due to accumulation of low‐density lipoproteins (LDL) in the artery, resulting in plaque build‐up. LDL are phagocytized by macrophages which convert to foam cells, which leads to necrotic core plaque area. Macrophages exacerbate the inflammation by secreting various inflammatory cytokines producing reactive oxygen species (ROS) and nitric oxide species. Targeting macrophages in reducing inflammation is an effective therapeutic strategy with targeted nanoparticles. Guo et al. explored the application of Naringenin (Nrg) loaded lipid PLGA based NPs labeled with FA to target atherosclerotic plaque. After transcellular transport of FA‐PLGA lipid NP to PP, the NPs were transported to the plaque via bloodstream by macrophages. The targeted NPs had shown 1.5‐fold greater than non‐targeted by fluorescence, predominantly due to active targeting of FA to epithelial cells. Targeted NPs had also reduced the necrotic core area (by 27%), glucose levels, low density lipoproteins, triglycerides, and cholesterol, and increased high density lipoproteins than non‐targeted NPs.^[^ ^50^ ^]^ Overall, a decrease in relative aortic lesion area after 12 weeks treatment with FA‐LNP/Nrg, as compared to other groups, indicates successful treatment for atherosclerosis.

Bindarit (inhibitor of monocyte chemoattractant protein‐1 [MCP‐1]) loaded YC to target atherosclerotic plaque was studied and has shown remarkable accumulation in the aorta. After M cell mediated transport of bindarit‐loaded YC to the PP, macrophages uptake the YC for translocation to the atherosclerotic plaque for targeted delivery. β glycan is an integral component in YC which targets dectin‐1 on macrophage and monocytes. The targeted NPs have shown a reduction in plaque size (by 33%), TNFα, IL‐1β, MCP‐1, and triglycerides when compared to non‐targeted group. The targeted group has also shown a reduction in cholesterol, LDL, and reduced macrophage levels, indicating good preventive effects in the plaque formation.^[^ ^88^ ^]^ In another study, Rapamycin (RAP)‐Indomethacin (IND) loaded polyethyleimine (PEI) YC microparticle was transported to the PP via M cell and targeted to plaque by the macrophage present in the PP by lymphatic system. The targeted group had shown remarkable decrease by 55% in plaque, macrophage, MMP9, TNF‐α, and interferon‐γ (INF‐γ) and higher collagen formation than the non‐targeted group, that is, RAP‐IND/PEI NPs.^[^ ^83^ ^]^

Of all the studies targeting atherosclerotic plaque, YC are generally transported via the M cell mediated pathway when compared to the use of PLGA lipid NP, owing to the size of YC as other routes involving TJs would not be possible over M cells which has phagocytotic capacity. These targeted approaches have demonstrated the possibility to target atherosclerosis via oral route, which opens the door to the effective and safe delivery of a wide range of treatments, including small molecule medicines, peptides, proteins, and nucleic acids. Targeted approach in atherosclerosis should be designed to enable low dosage targeted delivery to prevent the degradation for treatment of long‐term chronic diseases such as atherosclerosis and heart diseases.

### 3.2 Cancer

PP route and lymphatic system have also been employed to target drug to non‐GI tract tumors via oral administration. FCL/GPM MPs were developed to deliver Gefitinib (GEF) to lung tumor. After the transport of FCL/GPM‐GEF to PP by M cell mediated transport, the macrophages in the PP uptake FCL/GPM‐GEF due to the presence of β glycan that targets dectin‐1 present on macrophage, leading to the translocation to the tumor site for targeted delivery. The folate decorated NP targets the folate receptors which are overexpressed on tumor cells, leading to an enhanced accumulation of FCL/GPM in the tumor site than liposomes only (non‐targeted). The targeted microparticle (FCL/GPM) reduces tumor weight by 54% compared to non‐targeted NPs. Targeted microparticles have also shown higher tumor inhibition rate by 1.47‐fold than commercial GEF formulation.^[^ ^17^ ^]^

**Figure 7:**
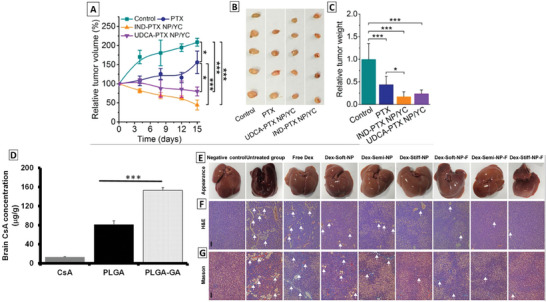
Therapeutic effects of various non‐GI diseases after oral targeted drug delivery. A–C) Therapeutic antitumor efficacy of PAX nano therapies delivered by UDCA‐NP/YC in MCF‐7 xenografts bearing mice, showing decrease in tumor for targeted group, proving to be effective for cancer treatment: reduced relative tumor volume in UDCA‐PAX NP/YC (A), appearance of the excised tumor showing a decrease in UDCA‐PAX NP/YC tumor size (B), and decreased relative tumor weight for UDCA‐PAX NP/YC (*P < 0.05, **P < 0.01, and ***P < 0.001) (C). Reproduced with permission.^[^ ^67^ ^]^ Copyright 2017, American Chemical Society. D) Cyclosporin A(CsA) kinetics showing higher CsA distribution (twofold increase) in brain for PLGA targeted with gambionic acid after 72 h in vivo after oral administration (***P < 0.001). Reproduced from the CC‐BY open access publication.^[^ ^23^ ^]^ Copyright 2016, Springer Nature. E–G) Liver fibrosis regression in SD rats: representative images of the effect of FcBP targeted PLGA lipid NP of different morphology (Stiff NP, Soft NP, and Semi NP) showing reduced collagen levels compared to Free Dex and untreated group for an effective treatment on liver fibrosis (E), H&E (F), and Masson trichrome staining; arrow heads indicate collagen fibers (G).^[^ ^52^ ^]^ Reproduced with permission under the terms of Copyright license.^[^ ^52^ ^]^ Copyright 2022, Elsevier.

YC assembled with ursodeoxycholic acid (UDCA), a natural compound of bile acids, is also used for the delivery of paclitaxel (PAX) to tumor and targeted with β‐glycan recognized by Dectin‐1 on macrophages. After M cell mediated transport, YC are ferried by macrophages to the neighboring lymphoid tissues due to the cytokine signals such as interleukins, TNF‐α which mobilize the macrophages to enter the blood stream. Various chemokines and cytokines such as monocyte chemoattractant protein 1 and macrophage colony‐stimulating factor are released by the tumor in attracting macrophages to the tumor, which migrate across the vascular endothelium in the targeted tumor site. The targeted YC microparticle shows a decrease in tumor weight by 50% and an increase in PAX concentration by 200% in the tumor site than free PAX group.^[^ ^67^ ^]^ **Figure** **7**A–C indicates reduced tumor size with UDCA‐PAX NP/YC after oral administration in mice bearing MCF‐7 breast cancer xenografts, proving to be effective for cancer treatment.

Various strategies have been developed to help NPs crossing intestinal epithelium to target non‐GI tract tumors. N‐trimethyl chitosan (TMC) with soy peptide aggregate (SPA or heated SPA [HSPA]) labeled with goblet cell targeting peptide, CSK, was developed to deliver vitexin (V) to tumor. In this system, Vitexin as a drug was encapsulated in HSPA/SPA, and then, covered with CSK‐TMC. CSK‐TMC with SPA and HSPA NPs evade the intestinal epithelial by paracellular means through the TJsand reach tumor site via blood circulation. However, the interaction between CSK peptide and TJs has not been studied in this research. Other studies have also shown that CSK peptide enhances uptake of nanoparticles and improves the bioavailability.^[^ ^89^ ^]^ The targeted group (particles with CSK‐TMC coating) has shown higher superoxide dismutase (SOD) and lower malondialdehyde (MDA) than non‐targeted (particles SPN‐V and HSPN‐V without CSK‐TMC coating) in liver and serum, indicating that targeted particles provided better protection of cells from oxidative stress.^[^ ^22^ ^]^

BU‐labeled PLGA lipid NP loaded with Sorafenib (Sor) and Salinomycin (Sal) for targeting hepatocellular carcinoma have shown better accumulation in the liver tumors and stronger anti‐tumor activity than the non‐targeted one. In order to cross the intestinal epithelium, the BU modified PLGA lipid NPs interact with monocarboxylate transport 1 (MCT1) present in the intestinal epithelium, leading to receptor‐mediated endocytosis to enter the bloodstream. The targeted NPs have shown reduced expression of various biomarkers for tumor progression such as glutathione (GSH), TGFβ, IL‐10, and increase in ROS, lipid peroxidation MDA, calreticulin (CRT), ATP, high mobility group box 1 protein (HMGB‐1), TNFα, 1L12, and increase in cell density of CD8^+^ T, CD4^+^T cells, and dendritic cell which correspond, respectively to enhanced anti‐cancer properties in liver tumors.^[^ ^19^ ^]^ Polyzwitterionic micelles made of poly(2‐[*N*‐oxide‐*N,N*‐diethylamino]ethyl methacrylate) copolymer with poly(ε‐caprolactone) were developed for delivery of PAX, an anticancer agent, and could effectively permeate through mucus, bind to the enterocytes, and then be transcytosed into systemic circulation by transepithelial transport via a nonlysosomal pathway. The micelles efficiently delivered PAX throughout the tumor tissues, leading to an antitumor activity reduced by 39%, compared to PEG counterparts.^[^ ^90^ ^]^

As discussed earlier, when using YC for targeted delivery, M cell mediated pathway is generally the most adopted method to cross intestinal epithelium due to the size of the microcapsule because other paths such as paracellular via TJs and transcellular would not be feasible. It is also an innovative method which does not require additional attachment of targeting proteins/aptamer as it always possesses β‐glycan recognized by Dectin‐1 on macrophages, which forms a natural targeting tool. Other delivery methods such as NPs synthesized with polymers (e.g., chitosan, PLGA), along with various moieties for targeting intestinal epithelium as described above, adopt various other transportation routes such as receptor mediated endocytosis and TJs opening for evading the intestinal barrier. Encouragingly, all the targeted delivery methods discussed here have shown positive therapeutic effects in treating cancer. Yeast microcapsule is one innovative method in drug targeting as it forms a biomimicking platform for oral delivery of chemotherapeutic drugs and other derivatives.

### 3.3 Neuronal Diseases

Neuronal diseases, including Alzheimer's and Parkinson's, are characterized by the deposition of amyloid beta (Aβ) peptide, degeneration of dopaminergic (DA) neurons, the formation of Lewy bodies within these neurons, and the accumulation of α‐synuclein.^[^ ^91^, ^92^ ^]^ Novel drug carriers need to be designed to be biocompatible, biodegradable, and able to cross the BBB. PLGA NPs are labeled with Tween 80 to deliver and target estradiol to the brain. After intestinal endocytosis, PLGA NPs enter the blood stream and reach the brain by crossing the BBB. However, the exact mechanism of crossing the BBB has not been studied. A higher estradiol level for NP labeled with 4% tween 80 has been shown to be 0.5‐fold higher than 1% tween 80 NPs, 1‐fold higher than non‐targeted; and reduced expression of amyloid beta‐42 (Aβ42) immunoreactivity was observed in the hippocampus region of brain.^[^ ^93^ ^]^ PLGA NPs were also labeled with GA, which showed the ability to cross the intestinal barrier, reach the systemic circulation, and penetrate the blood–brain barrier by targeting the transferrin receptors present on the BBB epithelium. PLGA‐ GA NPs to deliver cyclosporine A (CsA) have shown increase in CsA concentration by 2‐fold than non‐targeted one (without GA) and by 15‐fold than the CsA group.^[^ ^23^ ^]^ Figure 7D indicates an effective distribution of CsA in the brain (2‐fold increase) after oral administration of PLGA‐GA as compared to non‐targeted groups in vivo.

The oral approach to target brain and neuronal diseases faces two barriers including crossing the intestinal barrier and penetrating BBB. Carrier nanoparticles should be designed to cross both barriers. Dual targeted strategy to cross both barriers could be designed consisting of two different targeting/binding ligands and special focus should be placed on developing efficient packaging to improve the bioavailability as this route is one of the longest routes of delivery. From all the reported studies, to target neuronal diseases via oral administration, polymeric nanoparticles have been vastly explored as these nanoparticles are efficient in crossing the intestinal and brain barriers to reach the targeted site.

### 3.4 Other Non‐GI Diseases/Other Applications

Oral immunization or oral vaccine delivery can induce protective immunity in mucosal area for the treatment of intestinal infections and autoimmune diseases. Several studies have indicated that oral delivery vaccines have unique effects on intestinal infection because the primary immune cells tend to be home to effective site where antigen presenting cells (APCs) are first initiated.^[^ ^94^, ^95^ ^]^ Further, oral administration—the most popular natural medication delivery method—has the potential to immunize a large population against needle‐associated hazards and greatly increase patient compliance. Mannosylated chitosan (M‐CS) NP decorated with mannose and Eudacrit (Eud) used to deliver BSA for vaccine delivery. After M cell mediated pathway, M‐CS NPs were delivered to the immune system and had shown an increase in IgG levels by 1‐fold than Eud coated NP and 7‐fold increase than mannose coated NP. An increase in IgA levels by NP labelled with Eud and mannose by 0.42‐fold than Eud coated NP and 1‐fold than Mannose coated NP was also observed.^[^ ^81^ ^]^

YC is mainly composed of beta glycans and the potential anti‐inflammatory effects of beta‐glucans are thought to be related to their ability to interact with the immune systems, which are the targeting strategies for inflammation sites. They may modulate immune responses, potentially reducing inflammation. YC loaded with Indomethacin (IND) for treatment of acute paw inflammation has shown a reduced degree of inflammation by 44% and higher IND content by 300% than IND group.^[^ ^67^ ^]^

Female infertility is caused due to various factors such as aging, delayed childbearing, and inappropriate diet. FSH, which is responsible for oogenesis and stimulates ova production, is usually administered parenterally to treat female infertility. As FSH is secreted by the pituitary gland which binds to the FSH receptor on ovaries and stimulates ovulation, effective delivery strategies need to be employed to prevent gastric degeneration and improve FSH bioavailability. Nanostructured lipid carriers (NLCs) tagged with RGD peptide for targeting β1 integrins of M cells present in the PP to enhance lymphatic permeability to deliver FSH were explored by Raut et al.

NP labeled with RGD demonstrated enhanced penetration into PP than non‐targeted group based on ex vivo imaging analysis performed on rat's intestinal segments. In addition, accumulation and enhanced levels of FSH in the blood serum and increased weight of ovaries of rat model further corroborated the improved delivery of FSH to the ovaries with the targeted nanosystem.^[^ ^49^ ^]^

Yu et al. investigated the delivery of dexamethasone (Dex) for the treatment of liver fibrosis using lipid‐coated PLGA NPs of different morphology labeled with FcBP peptide, which target neonatal Fc receptor (FcRn) overexpressed in intestinal epithelium. After endocytosis and transcytosis by epithelial cells, the NPs were transported to the targeted hepatic chambers. FcBP‐labeled NPs have shown higher fluorescence by 2‐fold than the non‐labelled NPs, corresponding to the higher accumulation of labeled NPs in the liver. This also resulted in less collagen, lower alanine transaminase by 0.42‐fold, and lower aspartate aminotransaminase by 0.5‐fold than NPs without FcBP.^[^ ^52^ ^]^ Figure 7E–G indicates a reduced collagen in the liver after the treatment with FcBP modified NP in Sprague–Dawley rats proved to be effective for liver fibrosis and suggesting the excellent anti‐hepatic fibrosis effect. The untreated groups showed high level of collagen and a slight reduction of collagen for the Dex treated group.

The development of various nano formulations to achieve the desired results, along with good stability, targetability, and bioavailability for large molecules via oral delivery have been studied. The various delivery strategies, such as oral immunization, mannosylated chitosan NP, YC loaded with Indomethacin, and nanostructured lipid carriers tagged with RGD peptide, have shown positive effects in treating intestinal infections, autoimmune diseases, inflammation, and female infertility. These strategies have demonstrated increased immune response, reduced inflammation, enhanced targeting, and positive therapeutic effects in their respective applications. These advancements serve as a doorway for various applications such as vaccine deliver, drug delivery, and other applications for disease treatments. **Table** **3** tabulates all the various targeted deliveries of nanoparticles via oral administration for treating non‐GI diseases by intestinal targeting and diseased site targeting.

**Table 3:** Various targeted deliveries of nanoparticles via oral administration for treating non GI diseases by crossing gut barrier and targeting diseased site.

Material of particle	Active agent	Target disease, animal model	Strategy to cross the gut barrier	Strategy to target disease site	Characteristics of particle	Key therapeutic effects	Reference
Lipid‐PLGA based nanoparticles (LNP)	Naringenin (Nrg)	‐Atherosclerosis ‐ApoE knockout mice	Peyer's patches (PP) in GALT involving M cell mediated transport of LNP	Folic acid (FA) targets folate receptor distributed in the GI tract and macrophage of plaque	Size: 128.2 ± 5.8 nm Zeta potential (ZP): −1.04 ± 1.02 mV Morphology: core–shell like	FA‐labeled NPs loaded with naringenin reduced the atherosclerotic necrotic core area by 0.27‐fold than non‐targeted NPs. FA‐labeled NPs loaded with naringenin reduced the expression levels of glucose (0.2‐fold), low density lipoprotein (negligible change), cholesterol (0.14‐fold), and triglycerides (0.5‐fold); and increased the high density lipoprotein (0.4‐fold) compared to the non‐targeted NPs.	[50]
YC used as targeted group Polyethyleneimine (PEI) used as non‐targeted group	Bindarit (BIN)	Atherosclerosis ‐Apoe‐/‐ mice	PP translocation involving transport of YC by M cell mediated transport	β glycan present in yeast capsule, which is recognized by dectin 1 on monocytes and macrophage of plaque	Size: BIN/PEI NP: 28 ± 4.4 nm BIN/PEI‐YC: larger size (exact size was not reported) ZP: close to zero	Bindarit loaded YC has shown a reduction in plaque area by 0.33‐fold, a reduction in TNFα by 0.5‐fold, reduction in IL1β by 0.16‐fold, reduction in MCP1 by 0.16‐fold, and a reduction in triglycerides by 0.5‐fold as compared to non‐targeted NPs.	[88]
YC loaded with polyethyleneimine (PEI) nanoparticle was used as targeted group. Polyethyleneimine (PEI) nanoparticle used as non‐targeted group.	Rapamycin (RAP) Indomethacin (IND)	Atheroslerosis ‐ApoE−/− mice.	Transcytotic absorption in the GI tract via M cells in PP, followed by subsequent endocytosis in resident monocytes/macrophages, and final translocation through recruitment to diseased sites via the lymphatic system	β‐glucan, which targets Dectin‐1, is predominantly present on macrophage.	Size: 4.6 ± 0.6 µm ZP: −13.7 ± 0.4 mV	Rapamycin and indomethacin loaded in polyethyleneimine (PEI) nanoparticle encapsulated within YC have shown reduction in necrotic plaque area by 0.55‐fold than non‐targeted NPs.	[83]
Liposome loaded with Gefitinib and ZnO quantum dots, decorated with folate‐chitosan encapsulated inside β‐1,3‐D‐glucan porous YC	Gefitinib (GEF) and ZnO quantum dots	‐Target lung cancer ‐PC‐9 tumor‐bearing BALB/c nude mice for therapeutic study ‐Sprague‐Dawley rats for PP uptake and pharmacokinetic study	PP translocation involving transport by M cell mediated transport	Folate binds to folate receptors on many human epithelial tumor cell surfaces, but is rarely found on the normal cell surface. (Non‐targeted, i.e., without folate group not used)	Gefitinib loaded folate‐chitosan located liposome Size: 180.2 ± 13.1 nm ZP: 4.55 ± 0.68 mV Gefitinib and ZnO loaded liposomes Size: 142.3 ± 7.6 nm ZP: −7.44 ± 0.99 mV	Gefitinib loaded folate chitosan decorated liposome encapsulated within YC has shown a reduction in tumor weight by 0.54‐fold and 3.3‐fold than gefitinib folate chitosan decorated liposome and folate chitosan decorated liposome encapsulated in yeast, respectively.	[17]
YC loaded with polyethyleneimine (PEI) nanoparticle is used as targeted group. Polyethyleneimine (PEI) nanoparticle is used as non‐targeted group.	Paclitaxel (PAX)	‐Tumor ‐Sprague–Dawley rats with B16F10 (melanoma) or MCF‐7 (breast cancer) cells subcutaneously	Transport of YC by M cells in PP	β‐glycan recognized by Dectin‐1 on macrophages	Size: 4.7 µm ZP: −6.5 mV	Paclitaxel loaded polyethyleneimine (PEI) nanoparticle in YC showed reduced tumor weight by 0.6‐fold and an increase in paclitaxel by 4.7‐fold than non‐targeted NPs.	[67]
Poly(2‐[*N*‐oxide‐*N*,*N*‐diethylamino]ethyl methacrylate) (OPDEA) ‐ poly(ε‐caprolactone)(PCL)	Paclitaxel (PAX)	‐Targets liver cancer ‐ 4T1 xenograft mouse model in 8‐week‐old female BALB/c mice	Caveolae‐ and macropinocytosis‐related pathways		OPDEA‐PCL/PAX nanoparticle Size: 45.4 ± 3.1 nm ZP: −5.6 ± 1.4 mV	Paclitaxel loaded OPDEA‐PCL/PAX nanoparticle for targeting liver cancer has shown higher bioavailability, reduced tumor volume by 1.3‐fold, and inhibited the growth of tumor cells as compared to PEG‐PCL/PAX	[90]
PLGA nanoparticle	Estradiol(E2)	‐Targets Alzheimer's ‐ Ovariectomized (OVX) rat model of Alzheimer's disease (AD) Sprague–Dawley (SD) that mimics the postmenopausal conditions	Intestine endocytosis	Tween 80 (T‐80) targeting astrocytes in the brain (receptor in brain not given)	Estradiol PLGA nanoparticle Size: 134.7 ± 1.4 nm ZP: 68.5 ± 2.7 mV Estradiol loaded and coated with 1% tween 80 on PLGA nanoparticle Size: 134.7 ± 1.4 nm ZP: 68.5 ± 2.7 mV Estradiol loaded and coated with 4% tween 80 on PLGA nanoparticle Size: 157.0 ± 3.3 nm ZP: −3.6 ± 0.6 mV	Estradiol loaded and coated with tween 80 on PLGA nanoparticle for targeting Alzheimer's has shown a higher brain estradiol level than non‐targeted NPs. Estradiol loaded and coated with 4% tween 80 on PLGA nanoparticle has shown higher brain estradiol level by 0.5‐fold than Estradiol loaded and coated with 1% tween 80 on PLGA nanoparticle and 1‐fold than Estradiol loaded on PLGA without tween 80 coating. Estradiol loaded and coated with 4% tween 80 on PLGA nanoparticle showed reduced expression of amyloid beta‐42 (Aβ42) immunoreactivity in the hippocampus region of brain by immunohistochemistry.	[93]
Poly(lactide‐co‐glycolide) (PLGA) NP	Cyclosporine A (CsA), model peptide	‐Used for brain diseases ‐Male Sprague–Dawley rats (The rats did not have brain disease but the release level of CsA in the brain was measured)	Intestinal absorption	Gambogic acid (GA) binds to transferrin receptors in neuron and glial cell	Size: ≈110 nm ZP: ≈25–30 mV	Cyclosporine A loaded in PLGA nanoparticle coated with gambogic acid for targeting brain diseases has shown an increase in CsA concentration by twofold than non‐targeted NPs.	[23]
Mannosylated chitosan (M‐CS) nanoparticle	Bovine serum albumin (BSA)	‐Target immune cells for vaccine delivery in GALT ‐Sprague–Dawley rats (SD rats, 12‐week‐old, male) for targeting study and BALB/c mice (6–8 weeks old) for oral immunization study	Transport of Mannosylated chitosan nanoparticle by M cells in PP	Eudacrit (Eud), used for intestine targeting The mannose receptors are expressed widely in macrophages and dendritic cells.	Mannosylated chitosan nanoparticle Size: 313.1 ± 20.7 nm ZP: 40.8 ± 3.4 mV Bovine serum albumin loaded Mannosylated chitosan nanoparticle Size: 355.6 ± 25.3 nm ZP: 35.5 ± 5 mV Bovine serum albumin loaded and Eudacrit coated mannosylated chitosan nanoparticle Size: 558.2 ± 35.6 nm ZP: 12.3 ± 3.2 mV	Bovine serum albumin loaded and Eudacrit coated mannosylated chitosan nanoparticle to target immune cells for vaccine delivery has shown an increase in IgG levels by onefold than non‐targeted NPs and sevenfold increase than bovine serum albumin loaded mannose coated chitosan NPs (without Eudacrit coating) An increase in IgA levels by NPs labeled with both Eud and Mannose by 0.42‐fold than only Eudacrit coated chitosan NPs and 1‐fold than only mannosylated chitosan NPs was noted.	[81]
YC loaded with polyethyleneimine (PEI) nanoparticle is used as targeted group. Polyethyleneimine (PEI) nanoparticle is used as non‐targeted group.	Indomethacin (IND)	‐Acute paw inflammation Sprague–Dawley rats bearing Walker 256 carcinoma ‐BALBC mice macrophages (used for cellular uptake studies)	Transport of YC by M cells in PP	β‐glycan recognized by Dectin‐1 (beta glycan is a part of the YC) on macrophages	Size: 4.7 µm ZP: −6.5 mV	Indomethacin loaded polyethyleneimine (PEI) nanoparticle in YC has shown reduced inflammation degree by 0.44‐fold and higher IND content by 3‐fold as compared to non‐targeted nanoparticles.	[67]

## 4 Challenges and Obstacles

To attain therapeutic concentrations at the diseased site, enhanced effective NPs absorption is preferred. For effective treatment of various diseases and to reach the site, the intact NPs need to cross various barriers such as crossing the GI tract, entering the systemic circulation and remaining stable in the blood, and finally, reaching the targeted disease site.^[^ ^96^, ^97^ ^]^ Consequently, the targeting and absorption of NPs are constrained by numerous physiological barriers, presenting a significant challenge in drug development.

### 4.1 Instability of NPs

Stability, morphology, and drug release profile are important parameters for nanoparticle integrity and delivery to the effective targeted site. Small‐sized NPs have a large amount of surface area, which enhances the absorption into the GI tract due to improved interaction with the mucus barrier and reaching the diseased site such as atherosclerotic plaque, tumor, brain, and lymphatic vessels.^[^ ^98^ ^]^ However, NPs are also often found to be thermodynamically unstable, which encourages crystal formation and agglomeration.^[^ ^99^ ^]^ As discussed above, the study of lipid‐PLGA nanoparticles to deliver naringenin to atherosclerotic plaque has shown an aggregation after being stored for a prolonged period of 7 days in PBS at a physiological temperature of 37 °C, which leads to an obvious increase in size. A zeta potential (ZP) of 0 mV could also be a reason of aggregation. However, when stored at physiological temperature (i.e., 37 °C) in simulated GI conditions, it showed good stability without any change in encapsulation efficiency and particle size.^[^ ^50^ ^]^ GI conditions such as pH and temperature regulation could have an effect on nanoparticle stability and should be assessed. Stability also refers to the nature of the particles to efficiently load various drugs or medications within the delivery vehicle. For instance, YC may not be a suitable carrier for negatively charged nanoparticles due to the presence of phosphate and carboxyl groups on the yeast membrane, which cause electrostatic repulsion of negative charges. However, negative charged nanoparticles up to the size of 700 nm could still be efficiently packed into the YC after preincubation with cationic polymer polyethyleneimine. An inverse correlation between encapsulation efficiency and size of nanoparticle was observed whereby nanoparticles smaller than 700 nm would be suitable for enhanced encapsulation within YC.^[^ ^67^, ^83^ ^]^ Inspite of the large pores present in the YC resulting from bud scars, nanoparticles could be successfully entrapped within the YC. Drug leakage by these pores could be an issue during prolonged drug release and could lead to premature release of drug, decreasing the bioavailability. Hence, porosity of the YC is critical for efficient drug loading.^[^ ^18^, ^83^ ^]^

### 4.2 Fate of Nanoparticles in the GI Tract

The first line of defense for various external poisons and infections is the GI tract. To ensure that NPs reach the diseased site intact, they must remain stable within the GI tract.^[^ ^100^ ^]^ This presents a significant challenge for drug delivery. To avoid any damage of NPs in the harsh acidic environment, the choice of polymer such as PLGA, which can provide a sustained release in the acidic environment and avoid the enzymatic degradation, becomes a critical factor. Other approaches such as modification with mucoadhesive coating (PEGylation, chitosan) and enteric coating could resist the degradation in acidic environment, improving stability by preventing aggregation and providing resistance to mucus penetration; enzyme activity could be adopted in designing nanoparticle systems for GI tract.^[^ ^101^ ^]^ The mucus layer being a specialized membrane prevents various substances to enter the epithelial surface. Further, the NPs may get trapped within the mucus layer via electrostatic or hydrophobic interactions. NPs have either of the three fates after reaching the mucus layer, that is, excluding from the mucus layer, adhesion of NPs to mucus, or penetration of NPs through mucus. Penetration of NPs through mucus is extremely crucial to reach epithelial cells.^[^ ^102^ ^]^ The intestinal epithelium, the main transport barrier, must be overcome by NPs after they have successfully penetrated the mucus layer. The intestinal epithelium is reported to take up NPs by various pathways as discussed above such as paracellular, transcellular, transcytosis, and M cell mediated transport.^[^ ^103^ ^]^ An understanding of the mechanism of action has not been completely explored. Currently, transcytosis, transcellular uptake, and paracellular transport are the three uptake mechanisms that are understood.^[^ ^98^ ^]^ Various absorption methods might coexist, depending on the drug delivery technique. By modifying the NPs' physicochemical characteristics and using permeation enhancers to open up TJ to allow transport of drug by paracellular and transcellular methods or employing techniques based on receptor‐ and transporter‐mediated endocytosis, the intestinal absorption of NPs can be improved.^[^ ^20^, ^104^ ^]^

### 4.3 Challenges in the Blood Stream

After passing the GI tract barrier, NPs reach the systemic circulation and lymphatic system. NPs which are able to enter the lymphatic system, and then, into the blood circulation are able to bypass the presystemic hepatic first‐pass metabolism. As the biological milieu in blood is complicated, once NPs are exposed to blood, they may dissociate, resulting in payload leakage.^[^ ^105^ ^]^ Another issue with respect to transportation of YC loaded with drug to the effective targeting site, via the M cell pathway involving PP, is that the resident macrophages and monocytes in the PP need constant replacement with other macrophages and monocytes from the lymph nodes. Zhang et al. recorded that there is a decrease in the macrophage and monocytes after certain time points; they are not replenished by other splenic cells. This issue could eventually reduce the translocation of a certain percentage of the drug loaded YC; and hence, a smaller percentage proportional to the population of macrophage and monocyte would reach the targeting site. Moreover, if a large proportion of the drug loaded YC is present in the PP, premature drug dissolution may occur, particularly for drugs with pKa lower than the pH in the PP.^[^ ^67^ ^]^ An example is Indomethacin whose pKa is 4.5, which will tend to dissolve in alkaline pH present in the intestine. Therefore, if the indomethacin‐loaded YC is present in intestinal environment for prolonged time in slightly alkaline pH, premature dissolution of the drug will occur before reaching the target site. Further, the intact NPs would have two major challenges: 1) Protein adsorption and 2) interactions with phagocytes. This process seems to lower the amount of NPs that are available for accumulation in the intended location.^[^ ^106^ ^]^

### 4.4 Challenges in Reaching the Targeted Diseased Site and Therapeutic Effect

The targeted drug delivery reaches the targeted diseased site, which is a significantly small proportion. For targeting brain diseases, crossing the BBB is crucial and is responsible for hemostasis and only small molecules (lipid soluble with molecule weight less than 400 Da) can cross the BBB. Receptor mediated transcytosis and absorption mediated transcytosis are the two strategies to cross BBB.^[^ ^107^ ^]^ Although the best NPs formulation by intravenous delivery effectively penetrated the BBB by delivering up to 5% of the initial dose through intravenous injection, the efficacy of only around 0.1–0.3% of the initial oral dose was recorded for oral nanoparticle delivery to the brain.^[^ ^97^, ^107^ ^]^ PLGA NPs to deliver ergosterol to the brain via oral route for treating glioma with decreased heart and kidney distribution were reported. The BBB could not restrict the entry of PLGA NPs to the brain, which are consistent with other studies as well.^[^ ^108^, ^109^ ^]^ The exact mechanism is not reported yet. However, few studies suggested that PLGA nanoparticles might increase permeability and reduce P‐gp‐mediated efflux. Similarly, some nanoparticles can promote drug transport through the BBB by inhibiting active efflux transporters such as p‐glycoprotein.^[^ ^110^ ^]^

For targeting tumors, size and charge of the nanoparticle is a crucial parameter for accumulation in the tumor. Compared to larger nanomedicines, smaller ones can more effectively reach the tumor; however, they may also reach healthy tissue. Larger particles, on the other hand, have a considerably harder time dispersing in the tumor site.^[^ ^111^ ^]^ NPs in the 10–100 nm range are typically preferred because of their accumulation in the targeted tissues. In tumor tissue, positively charged particles tend to spread more evenly than negatively charged ones, whereas neutral NPs diffuse more quickly. The EPR effect in tumor is a common phenomenon where nanoparticles or drugs can accumulate in tumors. This effect causes the nonspecific accumulation of NPs is other organs with fenestration epithelial such as spleen, liver, and pancreas and results in unwanted toxicities.^[^ ^112^ ^]^ Therefore, further study on improving the tissue specificity and safety of oral gene therapy should be looked into for developing better therapeutics.

It was observed in rare cases that there were no significant differences in the therapeutic potential between free drug (i.e., IND) and IND NP/YC in the chronic inflammation model largely attributed to premature release of drug before the delivery system reached the inflammation site.^[^ ^67^ ^]^ Rigidity and stiffness of the NP system may have effect on its biodistribution. Stiff NP (e.g., NP containing PLGA packed in lipid shell) has shown significantly higher accumulation and distribution than soft NP (without PLGA core) in liver. The exact reason or mechanism how physical parameters such as young modulus, rigidity, and elasticity affect distribution of NP needs to be explored.^[^ ^52^ ^]^ This issue was also addressed in another study which showed that rigidity has a factor to play in pharmacokinetics and tissue distribution. Liposomes with a higher rigidity, which relates to higher cholesterol, have a longer elimination half‐life and higher tissue biodistribution.^[^ ^113^ ^]^ Designing a strategy for better therapeutic effect is crucial in relation to this issue.

## 5 Conclusion

In conclusion, the review comprehensively discussed the challenges and strategies associated with targeted oral nanoparticle/microparticle drug delivery for non‐GI diseases. The key goal had been to address the intricate physiological barriers that restrict the effective delivery of drugs to specific sites in the body. Strategies such as paracellular transport, endolysosomal escape, receptor‐mediated transport, and M cell‐mediated transport were examined in the context of targeting various diseases such as atherosclerosis, cancer, neuronal diseases, and other related applications. These techniques can overcome biological barriers and boost the efficacy of various medications, potentially opening new paths for treating such conditions.

However, several challenges and obstacles were identified throughout the review. These include the instability of nanoparticles, their fate in the GI tract, challenges in the bloodstream, challenges in crossing biological barriers. Studies for developing formulation strategies which utilize synthetic or natural targeted ligands to overcome these challenges generally focus on individual mechanism of drug delivery, bypassing the intestinal epithelium barriers and the difficulty in reaching the targeted diseased site to achieve therapeutic effect. Targeting ligands such as chitosan, FcBP peptide, and BU can potentially be engineered on nanoparticles for effective transcytosis depending on the choice of nanoparticle, size, and transport approach/route across the intestine. Developing targeting approaches for lesions outside the GI tract is still in its infancy, with many non‐GI targeted delivery methods relying on macrophage transportation in vivo. The precise route and mechanism of this transport remain unknown, necessitating further research. However, targeting the diseased site needs further understanding about the anatomy and receptors present on the targeting diseased site. Ligands with a strong affinity for the particular receptors need to be used for designing the nanoparticle system such as FA for targeting atherosclerosis and cancer. Physiological parameters of nanoparticles such as size, shape, surface charge, and rigidness are important parameters depending on the targeted diseases; and hence, the designing nanoparticle system.

This review highlighted the need for continued research and development to address these challenges and optimize targeted oral nanoparticle drug delivery for non‐GI diseases through oral administration. A dual targeting approach combining GI tract targeting and disease site targeting could potentially increase effectiveness and bioavailability. As drug delivery technology advances, future oral delivery systems are expected to target non‐GI cells and organelles with better effectiveness. Future research should focus on enhancing nanoparticle stability, investigating their fate in the GI tract and bloodstream and developing effective strategies to achieve therapeutic efficacy at targeted sites. Overall, addressing these challenges will play a critical role in advancing the field of targeted oral nanoparticle drug delivery for various non‐GI diseases.

## Conflict of Interest

The authors declare no conflict of interest.
